# Mitochondrial and Glycolytic Capacity of Peripheral Blood Mononuclear Cells Isolated From Diverse Poultry Genetic Lines: Optimization and Assessment

**DOI:** 10.3389/fvets.2021.815878

**Published:** 2022-01-28

**Authors:** Meaghan M. Meyer, Susan J. Lamont, Elizabeth A. Bobeck

**Affiliations:** Department of Animal Science, Iowa State University, Ames, IA, United States

**Keywords:** PBMC, cellular metabolism, glycolysis, mitochondrial respiration, genetic selection

## Abstract

Cellular metabolic preference is a culmination of environment, nutrition, genetics, and individual variation in poultry. The Seahorse XFe24 analyzer was used to generate foundational immune cellular metabolic data in layer, broiler, and legacy genetic strains using fresh chicken peripheral blood mononuclear cells (PBMCs). Baseline mitochondrial respiration [oxygen consumption rate (OCR)] and glycolytic activity [extracellular acidification rate (ECAR)] were determined in modern commercial laying hen (Bovans White) and broiler (Ross 308) lines, as well as the highly inbred lines of Iowa State University (L8, Fayoumi M-15.2, Spanish, Ghs-6), partially inbred broiler line, and advanced intercrosses of broiler by Fayoumi M-15.2 and broiler by Leghorn lines. Commercial broiler vs. Bovans layer and unvaccinated vs. vaccinated Bovans layer immune cell metabolic potential were compared following an in-assay pathway inhibitor challenge. Titrations consistently showed that optimal PBMC density in laying hens and broilers was 3 million cells per well monolayer. Assay media substrate titrations identified 25 mM glucose, 1 mM glutamine, and 1 mM sodium pyruvate as the optimal concentration for layer PBMCs. Pathway inhibitor injection titrations in Bovans layers and broilers showed that 0.5 μM carbonyl cyanide-4 phenylhydrazone (FCCP) and 1 μM oligomycin were optimal. Baseline OCR and ECAR were significantly affected by genetic line of bird (*p* < 0.05), with the dual-purpose, L8 inbred line showing the highest OCR (mean 680 pmol/min) and the partially inbred broiler line showing the greatest ECAR (mean 74 mpH/min). ECAR metabolic potential tended to be greater in modern layers than broilers (*p* < 0.10), indicating increased ability to utilize the glycolytic pathway to produce energy. OCR was significantly higher in vaccinated than unvaccinated hens (*p* < 0.05), while baseline ECAR values were significantly lower in vaccinated Bovans laying hens, showing increased oxidative capacity in activated immune cells. These baseline data indicate that different genetic strains of birds utilized the mitochondrial respiration pathway differently and that modern commercial lines may have reduced immune cell metabolic capacity compared with legacy lines due to intense selection for production traits. Furthermore, the Seahorse assay demonstrated the ability to detect differences in cellular metabolism between genetic lines and immune status of chickens.

## Introduction

Environmental and disease challenges alter feed intake, caloric requirements, and thus downstream energy metabolism in livestock. The impact of challenges on the immune system can be measured by isolating a population of cells and monitoring changes in cellular metabolism. Baseline cellular metabolic preference can be determined as impacted by disease status, nutrition, genetics, and individual variation in poultry. However, past research modeling energy usage in avian species has largely utilized immortal cell lines rather than heterogeneous cells isolated from whole blood collected from the animal itself. Hence, the objectives of the current study were to use the chicken as a model: fresh chicken peripheral blood mononuclear cells (PBMC) were used in an advanced metabolic assay to measure the effects of genetics and vaccination on baseline immune cell mitochondrial respiration and glycolysis. Furthermore, we aimed to quantify differences in the ability of cells isolated from two vastly divergent production strains (meat-type vs. egg-laying birds) to respond to an in-assay metabolic pathway inhibitor challenge. Before metabolic analysis, a series of titration experiments were conducted to ensure optimal plating conditions for cells assayed immediately post-isolation from whole blood because of the novelty of the cell types and treatments used.

Peripheral blood mononuclear cells (PBMCs) in chickens contain leukocytes, thrombocytes, and a small percentage of erythrocytes and monocytes (1) and are considered to be high-quality immune cells once isolated for use in further assays (2). Chicken cell culture lines have been used previously in the Seahorse metabolic assay, including immortal chicken macrophage-like cells (3), chicken embryo fibroblast cells (4), and cultured chicken primary brain cells (5), but to the knowledge of the authors, there is no published work using freshly isolated immune cells analyzed the same day. However, previous metabolic work has highlighted the functionality of immune cells as models for cellular metabolism (6, 7), hence, the relevance in using these cells directly after isolation. Chicken immune cells make for a particularly interesting model, as chicken populations in the last 60–70 years have been genetically selected from what were once multipurpose birds to two divergent, highly feed-efficient commercial lines: fast-growing broilers (8) or egg-producing layers (9). Ultimately, stringent artificial genetic selection for production traits of muscle accretion or egg production comes at the cost of other natural, biological functions that are also energetically expensive, i.e., the immune system (10). Baseline metabolic rates and immune cell pathway preferences between modern broiler and layer lines vs. legacy lines of chickens are, thus, of interest but have not been evaluated using an advanced metabolic assay. Furthermore, immune system activation, such as that induced by vaccination, has been shown to come at a cost in terms of redirecting energy from metabolism, nutrient partitioning, behavior, thermoregulation, etc. (11).

The Seahorse Extracellular Flux Analyzer (Agilent, CA, USA) is considered the “gold standard” for quantifying mitochondrial function and bioenergetics in cells. It measures two key outcomes: oxygen consumption rate (OCR) and extracellular acidification rate (ECAR), and includes assays specifically designed to stress cells and measure metabolic potential (Agilent). Mitochondria are organelles containing a double membrane, termed the outer and inner mitochondrial membranes, which, in turn, give rise to two components called the intermembrane space and the matrix (12). Passage of electrons through the electron transport chain creates energy, and the energy created by the electron transport chain establishes the proton motive force (12). In the Seahorse assay environment, without limited substrate availability, mitochondrial oxygen consumption is driven solely by proton motive force. Proton motive force is potential energy in the form of an electrochemical proton gradient that exists across the inner mitochondrial membrane and drives ATP production from ADP (13), and is increasingly used with increasing energy demands of the cell, hence, increasing the rate of oxygen consumption. When all ADP available has been converted into ATP by ATP synthase, oxygen consumption slows and is driven by proton leak. Mitochondrial proton leak is the process through which protons return to the mitochondrial matrix in the absence of ADP (14).

Oligomycin is an ATP synthase inhibitor that prevents phosphorylation of ADP, hence, slowing OCR and leaving only proton leak or non-mitochondrial respiration (14, 15). Glycolysis is then stimulated to meet the need of the cell for energy production (15). Glycolytic rate is measured through increased proton concentration/decreased pH, termed ECAR in-assay (Agilent). Carbonyl cyanide-4 phenylhydrazone (FCCP) is a mitochondrial uncoupler that depolarizes mitochondria, maximizing proton leak and, hence, oxygen consumption. After FCCP addition, the electron transport chain is no longer reliant on maintenance of a membrane potential, effectively speeding the passage of electrons to the maximum, limited only by substrate and oxygen availability (15). The Cell Energy Phenotype Assay (Agilent, CA, USA) allows assessment of the relative use of each energy-producing metabolic pathway and a unique comparison of the preference of the cells or more utilized pathway. After measuring baseline OCR and ECAR a pathway inhibitor challenge is introduced through simultaneous injection of FCCP and oligomycin. As described, FCCP is an uncoupler that disrupts mitochondrial membrane potential and drives oxygen consumption, while oligomycin inhibits ATP synthase and reduces mitochondrial respiration, hence, driving compensatory glycolysis. The effect of both stressors injected together is an increased glycolytic rate due to inhibited mitochondrial ATP production (Oligomycin) and increased oxygen consumption as the depolarized mitochondrial membranes drive the mitochondria to work to re-establish membrane potential (FCCP). Assay output includes baseline and stressed rates, metabolic rates, used together to calculate metabolic potential. In other words, “the cells' ability to meet an energy demand *via* respiration and glycolysis” (Agilent).

Therefore, the current work aimed to conduct titration experiments utilizing chicken PBMCs isolated from whole blood collections for same-day analysis in the Seahorse Xfe24 Analyzer in order to compare baseline metabolic phenotype between modern commercial broiler and layer lines as well as multiple inbred lines of chickens, some dating back to 1925. Additionally, the study aimed to determine metabolic response to in-assay pathway inhibitors between the two commercial lines, and to determine the effects of an *in vivo* immune system challenge in the form of a vaccine on laying hen PBMC metabolism, both baseline and following an in-assay pathway inhibitor injection.

## Materials and Methods

All live bird procedures were approved by the Iowa State University Institutional Animal Care and Use Committee (IACUC #8-16-8294-GM).

### Animals

Nine different chicken genetic lines from an existing colony at the Iowa State University Research and Teaching Farm (Ames, IA, USA) were used as the animal model, including two modern commercially available lines (laying hen and broiler) as well as seven additional genetic bird lines unique to Iowa State University. Birds were selected at random for blood draws, with the exception of a subset of recently vaccinated Bovans layers (IL-4 peptide vaccine; *n* = 15 hens) utilized for a bird-level immune challenge comparison. The commercial laying hen line used was Bovans White (Hendrix Genetics). Bovans layers were singly housed in 10 by 16-in. hanging cages (18 in. height) and were aged ~40 weeks at the time of the experiment. Approximately 140 Bovans laying hens were available for blood collections throughout this experiment. The commercial line of broilers (meat-type chickens) utilized were mixed sex Ross 308 (Aviagen), aged 5–7 weeks. Broilers were group housed in 4 by 4-ft pens of 10 (100 total broilers). The remaining genetic lines used for metabolic comparison were ~32 weeks of age and were singly housed hens kept in the same-sized cages as Bovans White hens, with the exception of the larger inbred broiler line, which were individually housed in 23 by 16-in. cages (21 in. height). There was a total of seven birds per genetic line available for blood draws. These lines are maintained by the Iowa State University for genetics research and include highly inbred lines (L8, Ghs-6, Spanish, Fayoumi M-15.2), a partially inbred broiler line, and two advanced intercrosses (broiler x inbred Ghs-6; broiler x inbred Fayoumi M-15.2). Brief genetic descriptions are provided in Table 1.

**Table 1:** Name and description of each of the genetic lines utilized for metabolic comparison with modern commercial layer and broiler lines.

**Line name**	**Description**
Line-8	Inbred since 1925. Laying hen body size
Spanish	Inbred since 1954. Originated in Spain
Fayoumi M-15.2	Inbred since 1954. Originated from Fayoum, Egypt
Ghs-6	Inbred since 1954. Originated from two US commercial Leghorn layer lines
Broiler (partially inbred)	Closed breeding population for 30 generations; accumulated inbreeding ~50%. Originated from commercial broiler parent line
Broiler x Ghs-6 advanced intercross	Advanced intercross line established from single broiler male and inbred Ghs-6 females
Broiler x Fayoumi M-15.2 advanced intercross	Advanced intercross line established from single broiler male and inbred Fayoumi M-15.2 females

### Blood Collection and Peripheral Blood Mononuclear Cell Isolation

Approximately 3 ml of blood/bird was collected from the brachial wing vein into a 3-ml syringe and transferred into sterile heparinized tubes (BD Vacutainer, NJ, USA). Peripheral blood mononuclear cells (PBMC) were isolated from whole blood using Histopaque 1077 and 1119 (Sigma-Aldrich, MO, USA). Live cells were resuspended in Seahorse assay media (pH 7.4, 37°C) and counted using a hemocytometer and trypan blue staining.

### Metabolic Analysis

Metabolic analyses were conducted on live primary peripheral blood mononuclear immune cells using the Cell Energy Phenotype Test within the Seahorse XFe24 Analyzer (Agilent, CA, USA). The Cell Energy Phenotype Test measures both mitochondrial respiration through oxygen consumption rate (OCR; pmol/min) and glycolysis through lactic acid production/extracellular acidification rate (ECAR; mpH/min) before and after a metabolic pathway inhibitor challenge. Assay preparation and procedure as outlined by Agilent (Seahorse XF Cell Energy Phenotype Test Kit User Guide) were followed to carry out titrations and metabolic tests using Xfe 24-well plates. Sensor cartridges were hydrated using Seahorse XF Calibrant, pH 7.4 (Agilent) the day before assay and placed in a 37°C incubator overnight. On the day of the run, assay media were prepared according to Agilent protocols and media optimization (described under *Titrations*) at pH 7.4 and placed in 37°C incubator prior to use. FCCP and oligomycin were resuspended in assay media and loaded into the Seahorse Sensor Cartidge Port A at a volume of 56 μl/port. Seahorse cell culture plates were prepared in advance with Cell-Tak solution (Corning, NY, USA) to better adhere cells to the plate. After live PBMCs were counted and resuspended in assay media, they were seeded in the Seahorse cell culture plate in duplicate at a volume of 100 μl/well and centrifuged at 200 × g for 1 min. Following centrifugation, fresh assay media were added to each well for a total volume of 500 μl/well. Four blanks/plate were filled with 500 μl of assay media alone. Cell culture plates were incubated at 37°C for 1 h prior to assay. Raw OCR and ECAR values are presented without an assay inhibitor challenge (baseline readings), and in the presence of an assay inhibitor challenge, the metabolic potential (%) is calculated by dividing the stressed (post-injection) values by the baseline values (pre-injection) × 100.

### Titrations

Due to the novelty of using fresh chicken cells isolated from whole blood within the Seahorse assay, prior to assessing challenges or differences between strains, a titration experiment was carried out using laying hen (Bovans white) and broiler (Ross 308) PBMCs to determine optimal cell seeding density, concentration of substrates in the assay media, and FCCP injection concentration. Each set of titrations were conducted using cells isolated from the same bird to allow comparison without inter-bird variation.

#### Cell Seeding Density

Cell seeding densities of 2, 3, 4, and 5 million cells per well were assessed using both laying hen and broiler PBMCs. Visual analysis of cells under a microscope for distribution in a monolayer as well as mean baseline OCR and ECAR values were used to determine ideal plating density.

#### Assay Media

Working media for this assay include Agilent Seahorse XF Base Media, pH 7.4, (Agilent, CA, USA) with the addition of sodium pyruvate (Sigma-Aldrich, MO, USA), L-glutamine (Sigma-Aldrich), and glucose (Sigma-Aldrich). Therefore, consecutive titration experiments were conducted using laying hen PBMCs to determine the optimal concentration of each substrate added to base media. Bovans laying hen cells alone were initially used for substrate optimization to provide optimal, identical assay media. Analyses between genetic lines were verified to behave similarly, and therefore, assays were conducted using the same media compositions for metabolic comparison so outcomes were not impacted by differences in substrate availability. Agilent recommends a concentration of 1 mM sodium pyruvate, 2 mM l-glutamine, and 10 mM glucose in the assay; hence, these concentrations were used as a starting point. Glucose was titrated first, testing 5.5, 10, 25, and 50 mM glucose in combination with the recommended concentrations of sodium pyruvate (1 mM) and l-glutamine (2 mM). Sodium pyruvate was optimized second, using 0.5, 1, 2, and 4 mM sodium pyruvate in combination with the recommended concentration of l-glutamine (2 mM) and 25 mM glucose, the optimized concentration from the glucose titration. Last, 1, 2, 4, and 8 mM l-glutamine were tested using the optimized concentration of sodium pyruvate, 1 mM pyruvate, and 25 mM glucose. Optimal concentration of each substrate was determined by peak baseline OCR and ECAR values.

####  Drug Injection

FCCP injection concentration was optimized using ideal cell seeding density and assay media concentrations determined in previous titrations in laying hen and broiler PBMCs. FCCP was titrated independently at 0.125, 0.25, 0.5, 1, and 2 μM. Following FCCP optimization, FCCP was titrated in laying hen cells only in combination with oligomycin (kept constant at 1 μM) to ensure that ideal concentration did not change. Optimal FCCP concentration was determined by response post-injection challenge, hence, using peak stressed OCR and ECAR values.

### Baseline Metabolism

Following assay optimization for chicken PBMCs, comparison of baseline metabolic performance with no pathway inhibitor challenge was compared across the nine genetic lines of birds previously described. Baseline OCR and ECAR values were measured using the Cell Energy Phenotype Test without pathway inhibitor (FCCP and oligomycin) injections. This allowed for an unchallenged comparison of inherent differences between immune cells isolated from different strains of birds under identical conditions.

### Metabolic Challenges

#### Pathway Inhibitor (*In vitro*)

Following baseline comparisons, mitochondrial respiration and glycolytic rate were analyzed within the Cell Energy Phenotype Test following simultaneous FCCP and oligomycin pathway inhibitor injection. The stressed (post-injection) OCR and ECAR were divided by baseline (pre-injection) OCR and ECAR and multiplied by 100 to calculate metabolic potential (%) of the cells. Metabolic potential, hence, measures the capacity of the immune cells to rise to an increased energy demand. These values were compared between the two common commercial lines of birds (broiler vs. layer) to determine genetic effect on the use of mitochondrial respiration and glycolysis when the immune cells are presented with a metabolic challenge. Seven distinct legacy genetic lines maintained at Iowa State University were also compared in separate assays.

#### Vaccination Status (*In vivo*)

A subset of vaccinated Bovans laying hens (*n* = 15) were compared against 15 unvaccinated hens to study the effects of a practical immune challenge on both baseline OCR and ECAR as well as metabolic potential (mitochondrial and glycolytic). The vaccine administered contained IL-4 peptides conjugated to bovine gamma globulin. The first dose used Fruend's complete adjuvant and a booster, 3 weeks later, used Freud's incomplete adjuvant. Hens received 1 ml of oil emulsion vaccine subcutaneously across four sites (breast and legs). Blood was collected for Seahorse metabolic assay approximately 12 weeks following vaccination booster.

### Statistical Analysis

Immune cell metabolic data generated using the Cell Energy Phenotype Test (Seahorse XFe24 Analyzer) were analyzed using Proc Mixed, a mixed linear model, following assessment of data distribution and normality using Proc Univariate in SAS Version 9.4 (NC, USA). Titration data (compared within Bovans laying hen and Ross 308 broiler genetic lines only) were analyzed with the fixed effect of cell seeding density, media substrate concentration, and pathway inhibitor concentration alone. Following titration for optimal assay conditions, baseline ECAR and OCR values (nine genetic lines compared) and metabolic potential (%; commercial broiler and Bovans layer lines compared) were analyzed with the fixed effect of bird genetic line and using a *post-hoc* Tukey–Kramer adjustment for all pairwise comparisons. To study the effects of an *in vivo* bird challenge within the commercial Bovans layer line only, OCR, ECAR, and metabolic potential data were analyzed with the fixed effect of vaccination status (unvaccinated vs. vaccinated). For all measures, least square means (LSMeans) and standard error (SEM) are reported; a value of *p* ≤ 0.05 was considered statistically significant.

## Results

### Titrations

#### Cell Seeding Density

**Figure 1:**
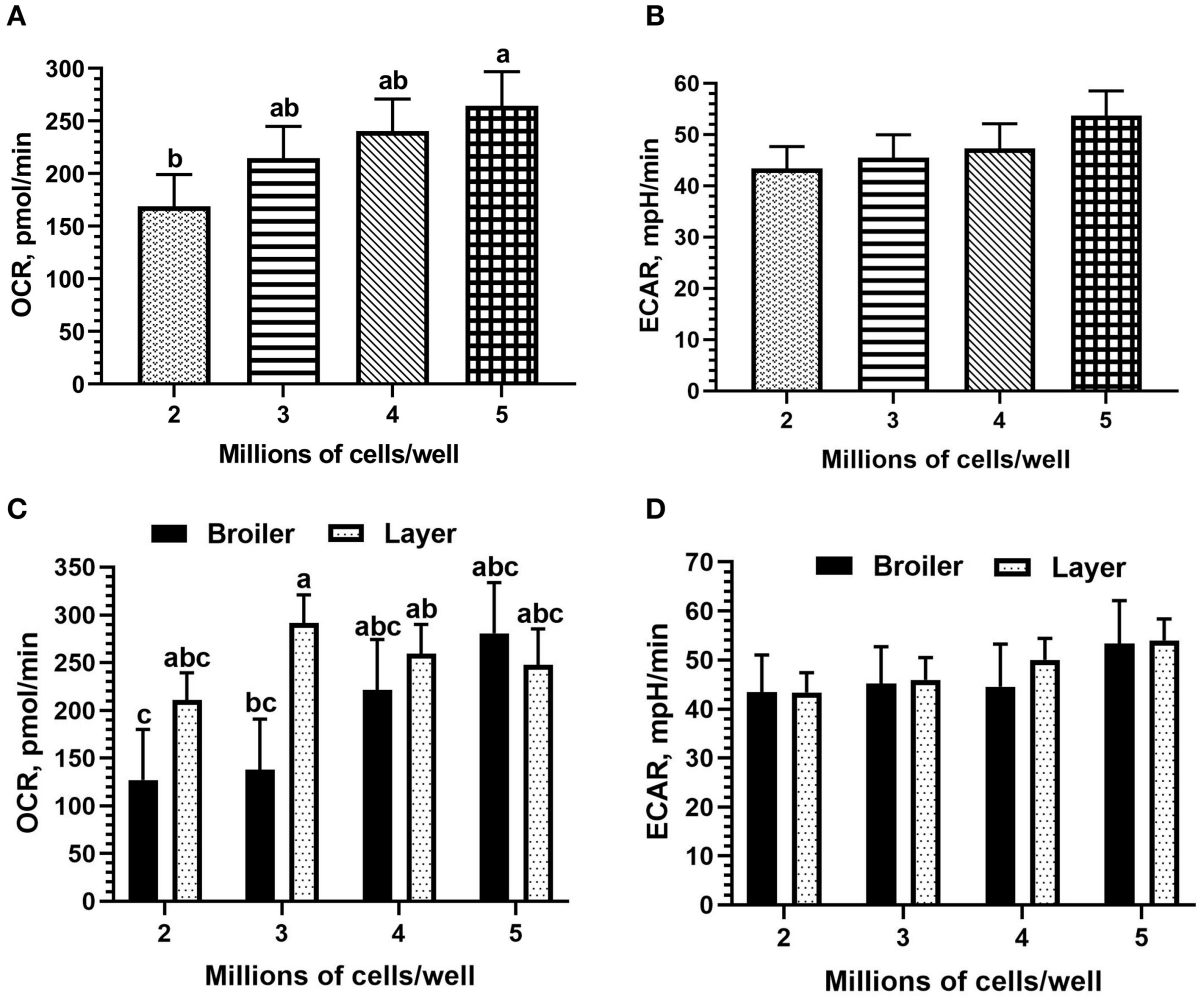
Cell seeding density titrations for optimal results using fresh chicken peripheral blood mononuclear cells (PBMCs) (Ross 308 broiler and Bovans layer) in the Seahorse Xfe24 Analyzer (Agilent) by main effect of cell seeding density on **(A)** oxygen consumption rate (OCR) and **(B)** extracellular acidification rate (ECAR), and by the cell seeding density x line of bird interaction on **(C)** OCR and **(D)** ECAR. All data are presented as least square means (LSMeans^1^) (SEM). ^1^Bars that do not share letters indicate means that are significantly different (*p* ≤ 0.05).

Cell seeding density did not affect mean OCR nor ECAR, but all titration values fell within an optimal range (OCR: 40–450 pmol/min; ECAR: 20–120 mpH/min). However, both OCR and ECAR values increased with increasing density as expected (Figures 1A,B); OCR was significantly increased between 2 and 5 million cells per well (mean 168.97 vs. 264.38 pmol/min). When including the effect of bird line (both broiler and layer PBMCs were used for seeding density titrations), line of bird significantly affected OCR (*p* = 0.05), broiler mean of 191.8 pmol/min vs. 252.39 pmol/min in layer, but not ECAR. Resulting bird type x seeding density interactions were not significant, but differences existed between broiler and layer seeding densities in mean OCR (Figures 1C,D). The peak OCR was observed at 3 million cells/well in Bovans layers (mean 291.43 pmol/min) and at 5 million cells/well in broilers (mean 280.87 pmol/min). Peak ECAR was observed at 5 million cells/well in both broilers and Bovans layers (broiler mean 53.35 pmol/min; layer mean 53.97 pmol/min). However, a key requirement of an accurate Seahorse assay is the maintenance of a monolayer of cells, visualized under microscope post-plating. Due to this requirement and the physical limitations of the 24-plate well size, 3 million chicken PBMCs per well were determined as the maximum without inducing overlapping cells. Taking this into consideration, along with the optimal OCR in layers observed at 3 million cells per well, 3 million cells were plated, moving forward, for all titrations and metabolic assays using chicken immune cells.

#### Assay Media

**Figure 2:**
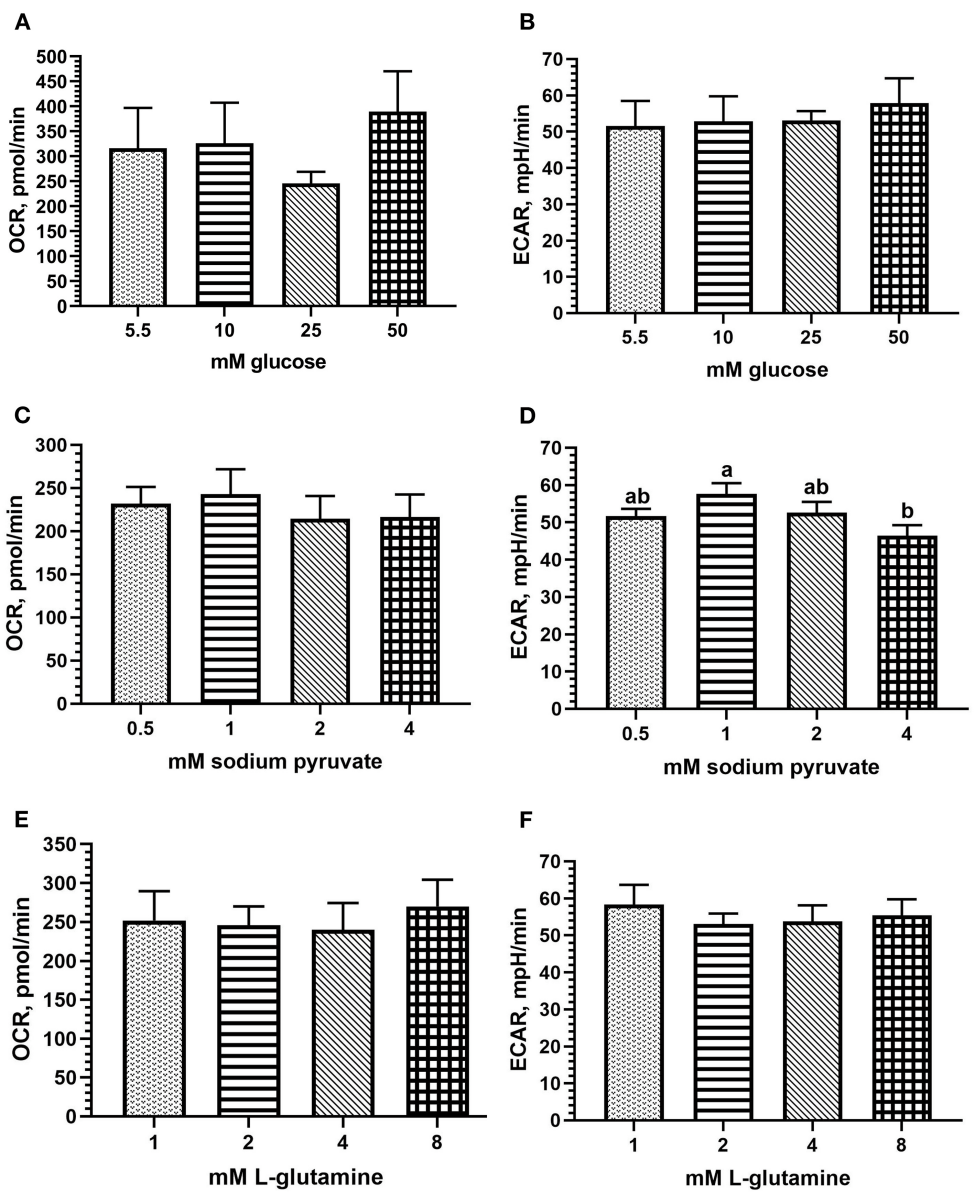
Assay media substrate titrations for optimal results using fresh chicken PBMCs in the Seahorse Xfe24 Analyzer (Agilent) by main effect of substrate concentration, including **(A)** glucose; OCR, **(B)** glucose; ECAR, **(C)** sodium pyruvate; OCR; **(D)** sodium pyruvate; ECAR; **(E)** l-glutamine; OCR; and **(F)** l-glutamine; ECAR. All data are presented as LSMeans^1^ (SEM). ^1^Bars that do not share letters indicate means that are significantly different (*p* ≤ 0.05).

The main effect of glucose concentration in the assay media was not statistically significant on OCR nor ECAR, but peak OCR (mean 389.38 pmol/min) and ECAR (mean 57.82 mpH/min) were each observed at 50 mM glucose concentration (Figures 2A,B). Due to lack of statistical significance, physiological limits, and the similarity between 25 and 50 mM in ECAR results (53.07 vs. 57.82 mpH/min), 25 mM glucose was determined adequate and used for the remaining titrations and metabolic assays. Sodium pyruvate concentration did not affect OCR but approached significance for ECAR (*p* = 0.08), with a peak of both values occurring at 1 mM pyruvate (mean 243.1 pmol/min and 57.65 mpH/min, respectively, Figures 2C,D). Hence, 1 mM was determined optimal for this substrate. The effect of L-glutamine concentration in the assay media was not significant on OCR or ECAR. Numerically, peak OCR was observed at 8 mM L-glutamine (mean 269.93 pmol/min; Figure 2E), and peak ECAR occurred at 1 mM L-glutamine (mean 58.32 mpH/min; Figure 2F). As 8 mM may exceed physiological relevance, and 1 mM followed in OCR peak (mean 251.82 pmol/min), 1 mM L-glutamine was determined optimal for the assay.

#### Drug Injection

**Figure 3:**
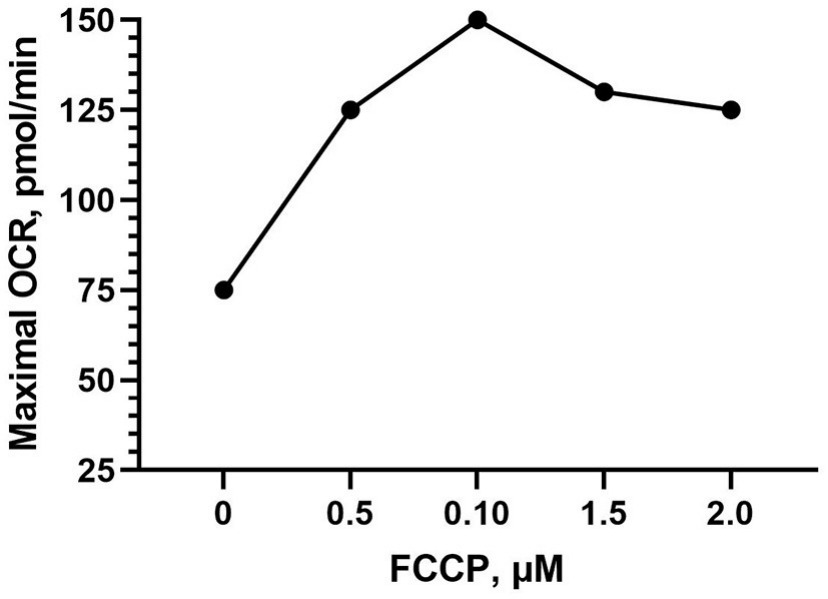
Example carbonyl cyanide-4 phenylhydrazone (FCCP) titration within the Seahorse Cell Energy Phenotype test showing the established effect of a plateau in OCR following peak respiration stimulation.

**Figure 4:**
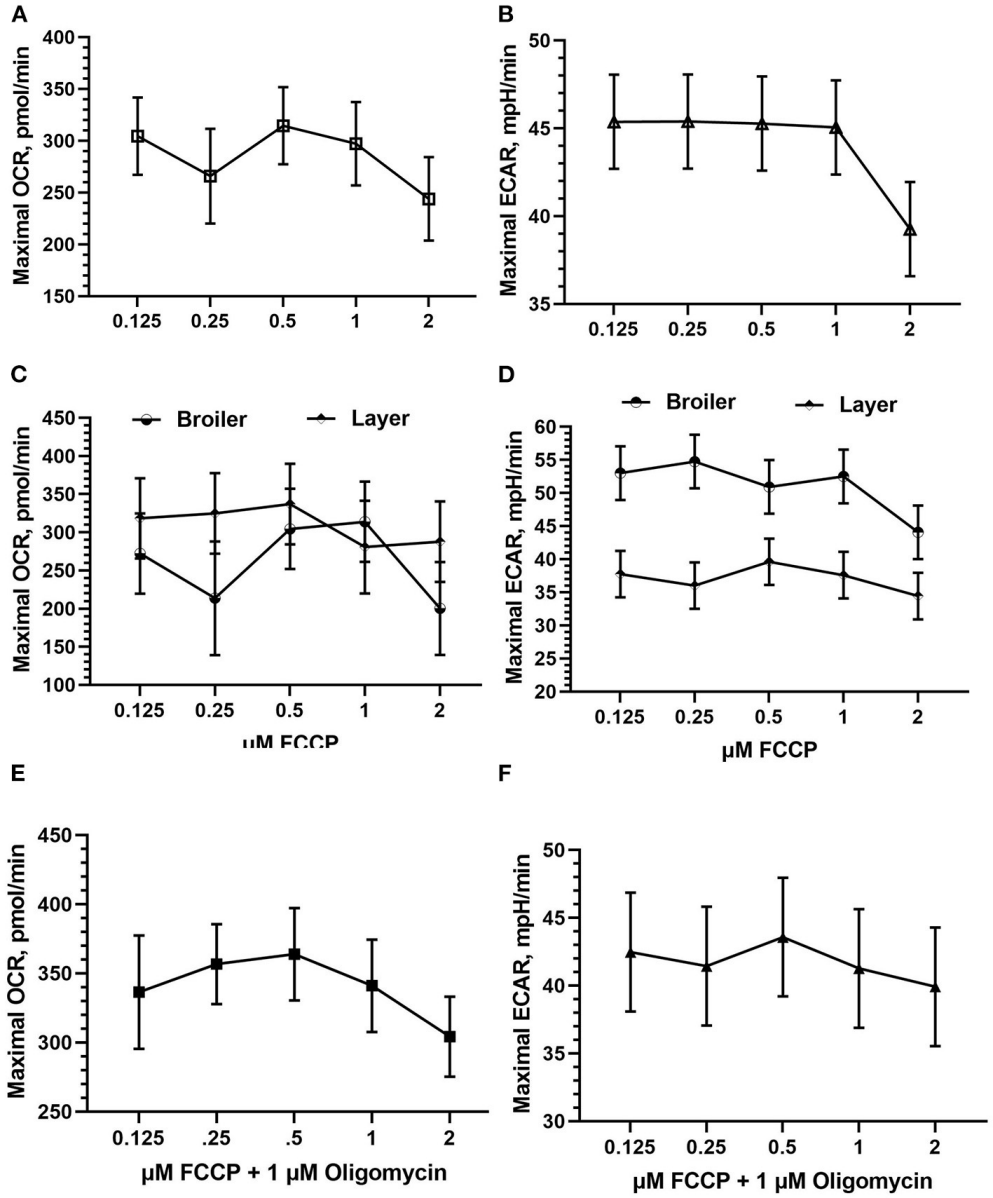
FCCP injection optimization using fresh chicken PBMCs in the Seahorse Xfe24 Analyzer (Agilent) by main effect of FCCP concentration on **(A)** OCR, **(B)** ECAR, and the interaction of FCCP concentration x bird line (Ross 308 broiler vs. Bovans layer) on **(C)** OCR and **(D)** ECAR. Last, FCCP was titrated in Bovans white layer cells in combination with 1 μM oligomycin on **(E)** OCR and **(F)** ECAR. All data presented are LSMeans (SEM).

FCCP was optimized in both Bovans layer and broiler PBMCs. FCCP titration is based off of maximal OCR response and is established to reach a plateau following maximal respiration (Figure 3). The effect of FCCP concentration injection was not significant for OCR nor ECAR in the current study. Peak OCR occurred at 0.5 mM FCCP (mean 314.58 pmol/min; Figure 4A), and peak ECAR was nearly equivalent between 0.125 and 1 μM FCCP (Figure 4B). The bird line x FCCP concentration interaction was not significant for OCR nor ECAR. Peak OCR occurred at 1 μM FCCP in broiler cells (mean 313.78 pmol/min) and at 0.5 μM FCCP in layer cells (mean 337.01 pmol/min; Figure 4C). Peak ECAR occurred at 0.25 μM FCCP in broilers (mean 54.76 mpH/min) and at 0.5 μM FCCP in Bovans layers (mean 39.62 mpH/min; Figure 4D). When including oligomycin in the titration at a constant concentration of 1 μM in layer cells alone, 0.5 μM FCCP induced peak OCR (363.84 pmol/min; Figure 4E) and ECAR (43.58; Figure 4F). Due to consistency in 0.5 μM FCCP inducing optimal response in layer cells with and without oligomycin, and to the variability in the results from broiler cells, 0.5 μM was determined ideal and used moving forward in the metabolic assay.

### Baseline Metabolism

**Figure 5:**
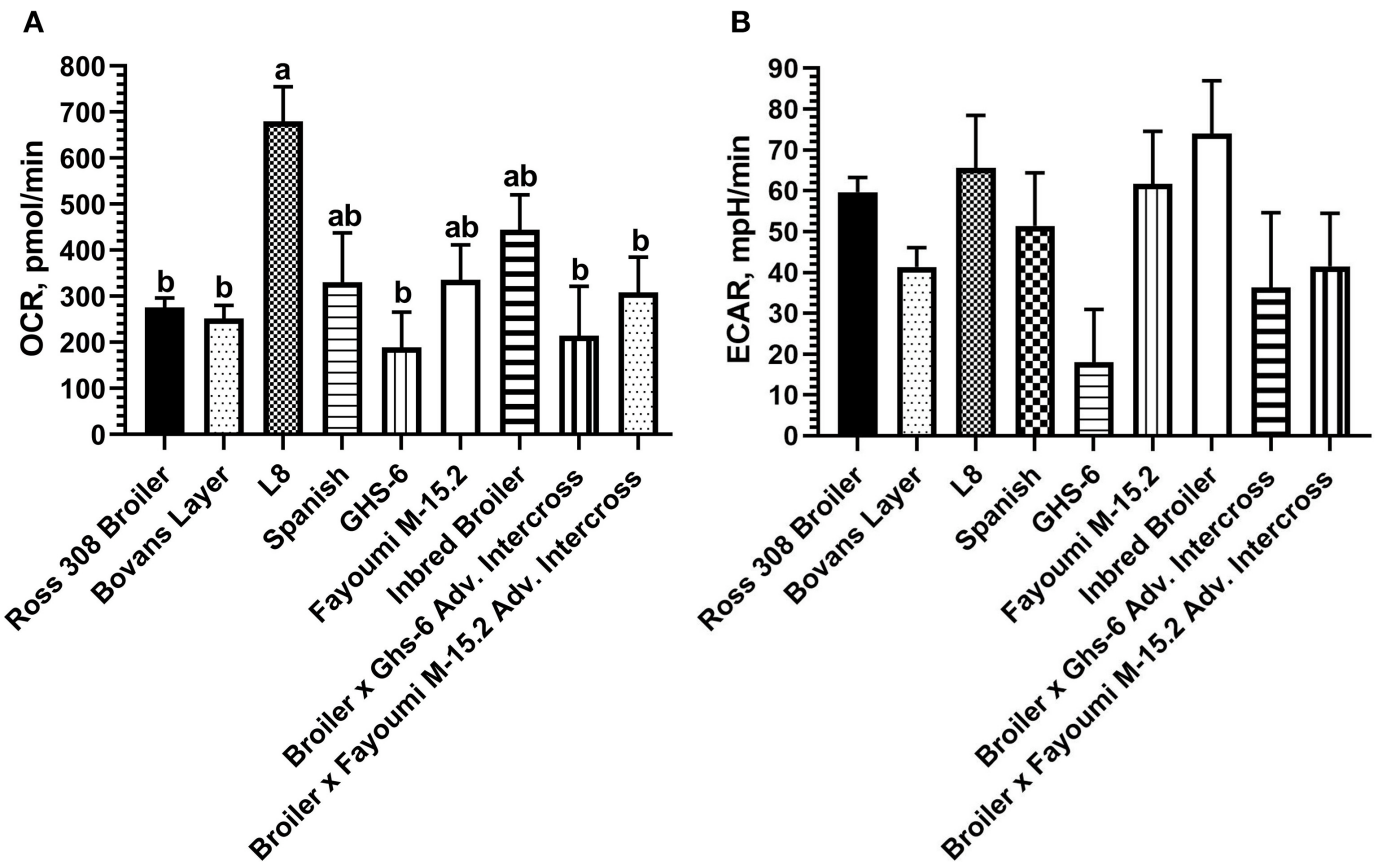
Baseline metabolic **(A)** OCR and **(B)** ECAR observed across nine strains of chickens using PBMCs within the Seahorse Xfe24 Analyzer with the main effect of bird genetic line. All data are presented as LSMeans^1^ (SEM). ^1^Bars that do not share letters indicate means that are significantly different (*p* ≤ 0.05).

Baseline OCR and ECAR values were compared within nine genetic lines of chickens using optimized conditions determined in laying hen and broiler PBMCs. The effect of genetic line was significant for baseline OCR (*p* < 0.01) and baseline ECAR (*p* = 0.01). Greater OCR was consistently observed in the L8 genetic line (inbred legacy line; mean 679.39 pmol/min) compared with all other lines (Figure 5A), representing a 428-pmol/min difference with the modern layer line, and a 404-pmol/min difference with the modern broiler line used here. Peak ECAR was observed in the partially inbred broiler line (mean 74.06 mpH/min), representing a 14-mpH/min difference with the modern Ross 308 broiler line, and a 33 mpH/min difference compared with the modern layer line (Bovan's white; Figure 5B).

### Metabolic Challenges

#### Pathway Inhibitor

Differences in metabolic potential (%) between broiler and layer PBMCs were examined using the combination of FCCP and oligomycin injection within the Cell Energy Phenotype Test. The effect of bird line was not significant on OCR metabolic potential, but Bovans layer cells (mean of 257.7%) showed a numerical increase of 50% using this mitochondrial pathway compared with broilers (mean 207.4%) when faced with the pathway inhibitor challenge. The effect of genetic line on ECAR metabolic potential approached significance (*p* = 0.08), with a 32.4% difference between layer (mean 207%) and broiler cells (mean 174.5%).

#### Vaccination Status

Vaccinated and unvaccinated layer PBMCs were first compared at the baseline to determine differences in resting metabolic rate due to a bird-level immune challenge. Vaccination did not affect OCR, but vaccinated Bovans laying hens (mean 247.72 pmol/min) showed a numerical 15.7 pmol/min increase compared with unvaccinated hens (mean 232.05 pmol/min). ECAR was also unaffected by vaccination, but in contrast to OCR, vaccinated Bovans laying hens (mean 44.77 mpH/min) showed a decrease of 8 mpH/min compared with unvaccinated hens (mean 52.81 mpH/min). In the in-assay pathway inhibitor challenge, OCR metabolic potential was unaffected by vaccination, but vaccinated hens (mean 281.1%) showed an increased response of 14.6% compared with their unvaccinated counterparts (mean 266.6%). ECAR metabolic potential was likewise unaffected by vaccination, but unvaccinated hens (mean 222.2%) showed a 4.4% increase compared with the vaccinated Bovans layers (mean 217.83%).

## Discussion

The Seahorse Analyzer provides a unique opportunity to measure metabolic activity in living cells, but due to variation in cell/cell lines used, titrations are critical to ensure optimal conditions. Multiple authors have performed titration experiments and published protocols in order to provide guidance to assay a wide variety of cells, including human cell lines (HK2) (16), isolated mitochondria from mouse liver (17), mouse skeletal muscle (18), and human skeletal muscle (19), as well as immune cells, including mice lymphocytes (20), mice T cells (21), and human T cells, and B cells (22). Studies using poultry cells, specifically, has included immortal cell culture lines, such as chicken macrophage-like cells (3), embryo fibroblast cells (4), and primary brain cells (5). More recent Seahorse optimization and disease research has been published using PBMCs isolated from humans (23, 24) and human cell lines (25), and research using laying hen PBMCs within the Seahorse assay has been recently published by the current lab group (26), but no optimization of chicken PBMCs has been published. Peripheral blood mononuclear cells are, however, suitable and well-published cells to use in immune cell metabolic and disease research (25), and are an ideal option for Seahorse analysis. Hence, the cell-seeding density (3 million cells/well), assay media substrate concentrations (25 mM glucose, 1 mM sodium pyruvate, and 1 mM l-glutamine) and FCCP injection concentration (0.5 μM) determined here are a suitable guide for future use of chicken PBMCs in the Seahorse Xfe24 Analyzer to maximize the quality of OCR and ECAR. It is well-established that there is an optimal FCCP injection unique to cells used in the assay to stimulate peak or maximal respiration (OCR) before a drop or a plateau in response (Agilent; example visualized in Figure 3). In our assays utilizing chicken PBMCs, 0.5 μM FCCP produced the maximal response OCR most consistently (Figures 4A–F).

Genetic influence on metabolism is evident at a whole-animal level when selecting for specific traits. For example, in chickens selected for divergence in abdominal fat pad by Dupont et al. (27), changes in liver metabolism were observed to favor lipogenesis and ultimately greater abdominal fat deposition. Meat quality-focused work in broilers has shown that divergent selection for breast ultimate pH at harvest resulted in differences in carbohydrate and protein metabolism, specifically by altering glucose storage and key enzymes in both the mitochondrial respiration and glycolytic pathways (28). Additionally, lipid metabolism is shown to be dysregulated in broilers affected by the woody breast myopathy, an unintended consequence of selection for increased breast yield (29). Genetic selection for egg-producing traits has profoundly impacted bone metabolism in layers (30). Environmental conditions have been shown to affect metabolism in caged vs. free-range layers by Zhang et al. (31), where meat quality-focused inosine monophosphate metabolism genes were differentially expressed based on housing. Environmental heat stress in chickens has a clear impact on metabolism, inducing uncoupling of oxidative phosphorylation in the mitochondria and resulting heat production (32). Therefore, the differences in baseline PBMC mitochondrial respiration and glycolysis due to genetic variation in chickens observed in the current study are not unexpected but have not been previously studied at the cellular level.

**Figure 6:**
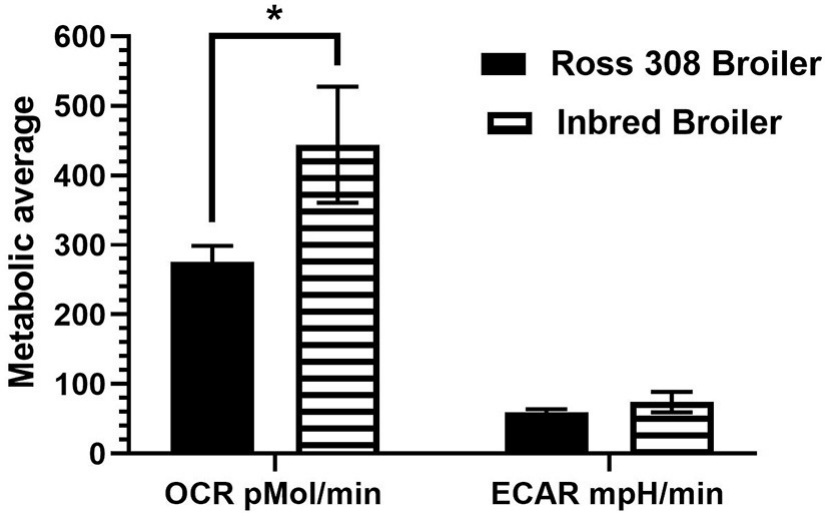
Baseline metabolic comparison of OCR and ECAR analyzed between two broiler genetic lines of different ages: Ross 308 broilers (aged 5–7 weeks) and inbred broilers (aged 32 weeks) using PBMCs within the Seahorse Xfe24 Analyzer with the main effect of bird age/genetic line. All data are presented as LSMeans^1^ (SEM). ^1^Asterisk indicates a difference in LSMeans approaching statistical significance (*p* < 0.10).

A novel feature of the current work was the comparison between two modern lines with seven unselected, inbred, or intercrossed lines of chickens, the earliest dating back to 1925. The significantly increased OCR, a measure of mitochondrial respiration, observed in the L8, dual-purpose line (inbred line since 1925) compared with modern commercial layer and broiler lines was an unanticipated outcome (Figure 5A) that may provide insight into consequences of intense genetic selection in modern commercial breeds. It has been established that selection for economic-focused traits in broilers has reduced diversity of immune system genetics (33) and that laying hens selected for feed efficiency show reduced antibody response to Newcastle disease virus (10). However, given that the L8 is highly inbred, its genetics is expected to be primarily fixed at the alleles in highest frequency at its founding. Overall, production traits are negatively correlated with disease resistance in poultry (34), so we may hypothesize that the unselected, dual-purpose L8 line has maintained more robust baseline immune cell energy production through mitochondrial respiration compared with the two modern day, high-producing strains. Interestingly, the inbred layer strain compared here (Ghs-6, inbred since 1954) was not different in baseline oxygen consumption compared with broilers and Bovans layers, which may be due to the specific genetic line (Leghorn). In terms of baseline glycolytic rate (measured by ECAR), no differences were detected between all nine genetic lines (Figure 5B). Regarding the effect of age of chickens on baseline metabolic capacity, the baseline OCR and ECAR were compared between the 5- and 7-week-old Ross 308 broilers and the aged, partially inbred broiler line (~32 weeks of age; closest genetic age comparison available). The effect of age between these two lines approached significance for OCR (*p* = 0.06) and did not affect ECAR (comparison in Figure 6). The lack of difference observed between ECAR in these lines indicate that the age of birds may not be a factor significantly impacting the glycolytic outcomes of this assay. However, OCR was increased in the aged line of inbred broiler, indicating a potentially increased capacity for mitochondrial respiration with age using this animal model. A future true metabolic comparison of the same genetic line of birds from different ages would be necessary to validate this point, as in the current study age and genetics of the bird were statistically confounded. Furthermore, the influence of sex has not been analyzed in this study, as the majority of the birds/genetic lines available for sampling were hens with the exception of mixed sex Ross 308 broilers. Future work comparing male and female cell populations within the same genetic line could explore or rule out this effect.

Glycolysis is considerably less efficient in terms of ATP production compared with mitochondrial respiration, producing only 2 vs. 36 molecules of ATP per molecule of glucose (35). Immune cells in a quiescent state generally rely on mitochondrial oxidative phosphorylation, while activated cells tend to transition to a glycolytic state to swiftly meet increased energy demands (36). Therefore, as the baseline comparison in the current work occurred in birds that were not disease challenged, it is likely that the glycolytic pathway was less preferred across all cell lines despite varying genetics. Notably, the partially inbred legacy broiler line showed numerically increased glycolytic rate, and the inbred Leghorn line (Ghs-6) showed a numerically decreased ECAR compared with all other lines. This variation may be due to reduced productive efficiency in the partly inbred broiler line, which appears to devote more resources to baseline immune cell metabolism, compared with the inbred layer line, which displayed consistently decreased OCR and ECAR. Interestingly, in the advanced intercross of these two lines (broiler x Ghs-6), both mitochondrial respiration and glycolysis were diminished compared with the inbred broiler line and seemed to be more influenced by the Ghs-6 immune cell metabolic phenotype. Overall, these data provide an interesting perspective in the shift of resource allocation away from the immune system toward performance characteristics with genetic selection over time.

This study also included introducing challenges, either applied to the bird itself or to immune cells within the metabolic assay. When commercial broiler and layer PBMCs were metabolically challenged using FCCP and oligomycin, no statistical differences were found in OCR metabolic potential, or the ability of the cell to rise to the inhibitor challenge using mitochondrial respiration. However, layer cells showed a numerically increased response of 50%, indicating a potentially biologically relevant change. In terms of glycolysis, Bovans layer PBMCs tended to show greater ECAR than broiler PBMCs, with a 32.4% increase. Broiler selection for weight gain has decreased immune system resources, an established phenomenon (10), likely to a greater extent than selection for high egg production has affected the immune response of the hen. Animal behavior may provide confirmation of this: broiler locomotion decreases over development; this is believed to be due in part to energy allocation toward significant weight gain that takes away from metabolic costs of standing, walking, perching, etc. (37). Laying hens, on the other hand, maintain significant physical activity throughout production (38). Additionally, antibody response in laying-type birds has been shown to be stronger and longer lasting than in broilers, making layers more suited for long-term humoral immune response (39). Therefore, a difference in metabolic potential of immune cells between these lines likely reflects their vastly different production traits and the resulting effects on immune system resources.

Due to the relatively more robust metabolic response in layer PBMCs, Bovans laying hens were used for a vaccine response experiment. Vaccines in poultry serve to activate a humoral immune response through antigen introduction (40). Vaccines stimulate B cells to produce antibodies specific to microorganisms of concern to prevent the spread of infection (41), hence, stimulating the immune system. The vaccine administered in the current study did not affect baseline OCR or ECAR, nor metabolic potential of either pathway. However, baseline and metabolic potential of mitochondrial respiration were numerically increased in vaccinated hens compared with unvaccinated hens, and baseline and metabolic potential of the glycolytic pathway in vaccinated Bovans laying hens were slightly numerically decreased in vaccinated hens. Stimulation of B cells has been shown to increase oxygen consumption and mitochondrial respiration (42), explaining the increased OCR in vaccinated hen cells both at the baseline and when challenged in-assay. This outcome also validates efficacy of the vaccine applied to hens in stimulating a humoral immune response. Additionally, the decrease in ECAR reflects the preference of the activated immune cells in the vaccinated model to use oxidative phosphorylation to produce energy rather than glycolysis, as stimulated B cells are not reported to induce glycolysis (42). These data indicate the ability of the Cell Energy Phenotype Test to detect differences in chicken PBMC as induced by a bird-level change in immune status.

In summary, the current work has established optimal assay parameters for use of fresh chicken PBMCs in the Seahorse Xfe24 Analyzer (Agilent) as well as validated use of the assay in determining metabolic differences among various genetic lines and vaccination status of the animal. A novel comparison of immune cell metabolism among modern commercial lines and inbred and intercrossed lines, one dating back to 1925, demonstrated shifts in immune system resources, showing a marked decrease in the baseline capacity of cells isolated from modern production broiler and layer lines. Finally, a vaccinated subset of Bovans layers demonstrated increased use of the mitochondrial respiration pathway, an indication of humoral immune response, compared with their unvaccinated counterparts. This optimized assay provides opportunity for future work using fresh poultry immune cells.

## Data Availability Statement

The raw data supporting the conclusions of this article will be made available by the authors, without undue reservation.

## Ethics Statement

The animal study was reviewed and approved by Iowa State University Institutional Animal Care and Use Committee (IACUC #8-16-8294-GM).

## Author Contributions

EB contributed to conception and design of the study. MM and EB contributed to on-farm sample collection, laboratory metabolic analysis, statistical analysis, and manuscript drafts. SL supplied birds and contributed to the manuscript content. All authors contributed to manuscript revision, read, and approved the submitted version.

## Conflict of Interest

The authors declare that the research was conducted in the absence of any commercial or financial relationships that could be construed as a potential conflict of interest.

## Publisher's Note

All claims expressed in this article are solely those of the authors and do not necessarily represent those of their affiliated organizations, or those of the publisher, the editors and the reviewers. Any product that may be evaluated in this article, or claim that may be made by its manufacturer, is not guaranteed or endorsed by the publisher.
